# Propensity-matched analysis of the effect of preoperative intraaortic balloon pump in coronary artery bypass grafting after recent acute myocardial infarction on postoperative outcomes

**DOI:** 10.1186/s13054-014-0531-z

**Published:** 2014-09-23

**Authors:** Pey-Jen Yu, Hugh A Cassiere, Sophia L Dellis, Nina Kohn, Frank Manetta, Alan R Hartman

**Affiliations:** Department of Cardiovascular and Thoracic Surgery, Hofstra-North Shore/LIJ School of Medicine, 300 Community Drive, Manhasset, NY USA; Department of Biostatistics, The Feinstein Institute for Medical Research, 350 Community Drive, Manhasset, NY USA

## Abstract

**Introduction:**

There is substantial variability in the preoperative use of intraaortic balloon pumps (IABPs) in patients undergoing coronary artery bypass grafting post myocardial infarction. The objective of this study is to determine the effect of preoperative IABPs on postsurgical outcomes in this subset of patients.

**Methods:**

From 2007 to 2012, 877 patients underwent isolated coronary artery bypass post myocardial infarction. Four hundred and six patients were propensity-score matched based on the likelihood of receiving a preoperative balloon pump. Total blood transfusion requirements, composite in-hospital morbidity and/or mortality end point, total hours in the intensive care unit, and length of hospital stay were compared between the two groups.

**Results:**

No significant differences in demographics, preoperative risk factors, intraoperative variables or length of hospital stay were found between patients with and without balloon pumps after propensity score matching. Compared to patients without balloon pumps, a higher percentage of patients with preoperative IABPs required transfusions. Patients with preoperative balloon pumps were more likely to have the composite end point of in-hospital morbidity (24.1% versus 12.8%, *P* <0.004), and increased hours in the intensive care unit (median hours: 69.0 versus 46.0, *P* <0.013) as compared to patients without balloon pumps.

**Conclusions:**

The use of preoperative IABPs in patients undergoing isolated coronary artery bypass grafting after myocardial infarction is associated with increased transfusion requirements, increased in-hospital morbidity and longer postoperative intensive care unit stay as compared to patients without IABPs.

## Introduction

Since their introduction in the 1960s, intraaortic balloon pumps (IABPs) have become the most widely employed circulatory assist device. IABPs are frequently used in high-risk coronary artery disease patients to augment cardiac output and increase coronary and visceral artery perfusion while awaiting myocardial revascularization. Accepted indications for preoperative insertion of IABPs in patients awaiting coronary artery bypass grafting (CABG) include hemodynamic instability, ongoing ischemia, poor left ventricular function, and/or critical coronary artery disease. However, there is substantial variability in the preoperative use of IABPs in patients undergoing CABG, especially in the setting of recent acute myocardial infarction. This is reflective of the lack of literature to support the effectiveness of preoperative IABP placement in this selective patient population and the increasing evidence against the benefit of IABPs in other clinical situations.

The benefits of IABPs in patients with acute myocardial infarction and those undergoing CABG surgery remain ill-defined. Acute myocardial infarction remains a significant predictor of preoperative IABP insertion in patients undergoing CABG, yet its utility in this patient population has not been adequately studied. The objective of this study is to elucidate the effect of preoperative IABPs on postoperative outcomes in patients undergoing isolated CABG after sustaining an acute myocardial infarction.

## Methods

This study was conducted with the approval of the North Shore-LIJ Health System Institutional Review Board. As this is a retrospective study utilizing de-identified data that was collected for the New York State and Society of Thoracic Surgeons (STS) databases, specific waiver of the need for individual patient consent was granted by the Institutional Review Board. Between 2007 and 2012, 877 consecutive patients within the North Shore-LIJ Health System underwent isolated CABG surgery within three weeks of an acute myocardial infarction (defined as cardiac biomarkers exceeding the upper limit of normal with a clinical presentation that is consistent or suggestive of ischemia). Decision for insertion of an IABP was at the discretion of the cardiologist or surgeon. Indications for preoperative IABP insertion include hemodynamic instability, medically refractory low output state and/or ischemia, and critical coronary anatomy. Aside from the minority of emergent cases, all preoperative IABPs were placed more than 24 hours prior to surgery. Unless contraindicated, intravenous unfractionated heparin was started within 24 hours after placement of the IABP. The decision to transfuse was at the discretion of the critical care physician and/or operating surgeon. Departmental transfusion guidelines allow transfusions for hemoglobin less than 8.0 g/dL regardless of advanced age and/or comorbidities, acute blood loss (30% of blood volume), and/or rapid blood loss without immediate control. Deviations from the guidelines must be justified by the attending surgeon and/or critical care physician. Patient data was collected prospectively in accordance to the New York State Department of Health Cardiac Surgery Reporting System and STS Adult Cardiac Surgery Database. Preoperative variables including patient demographics, comorbidities, preoperative medications, coronary and cardiac valvular disease burden, and preoperative hemodynamic status as well as intraoperative variables were collected on the day of surgery. Postoperative variables, including complications, were collected after the patient’s discharge from the index admission. All collected data was validated with the patient’s medical records by departmental clinical data coordinators after discharge. Data collected for the New York State Cardiac Surgery Reporting System is subject to independent auditing by the New York State Department of Health. All patients arriving in the operating room for CABG with an IABP in place were placed in the preoperative IABP group regardless of when the IABP was inserted. Definitions used for the preoperative risk factors and perioperative complications are standardized based on published guidelines by the New York State Department of Health for the New York State Cardiac Surgery Reporting System and the STS cardiac surgery database. In particular, preoperative hemodynamic instability is defined as the requirement of pharmacologic or mechanical support to maintain systolic blood pressure >80 mmHg or cardiac index during the immediate preoperative period. The presence of an IABP in and of itself does not place the patient in the hemodynamically unstable category unless the IABP was placed for hemodynamic support. It is not our center’s practice to place an IABP for the sole purpose of increasing the cardiac index to >2.0 liters/min/m^2^ unless there is hypotension or systemic manifestations of a low output state. Sepsis is defined by the presence of a Systemic Inflammatory Response Syndrome resulting from suspected or proven infection. Preoperative anticoagulation is defined as the use of intravenous or subcutaneous thrombin inhibitors within 48 hours of surgery. Gastrointestinal complication is defined as gastrointestinal bleeding, perforation, or infarction. Postoperative respiratory insufficiency is defined as failure to wean from the ventilator within 24 hours postoperatively. STS surgical risk scores were calculated using the online risk calculator [1]. To determine the baseline perioperative risk independent of the presence of an IABP for each individual patient, a modified STS risk score was calculated in which the presence of an IABP was not factored into the calculation.

Out of the 877 patients, 346 received an IABP preoperatively and 531 did not receive an IABP preoperatively. Five patients (two with an IABP and three without an IABP) had missing data and were excluded from further analysis. Of the 872 patients with complete data, 406 were propensity-score matched based on the likelihood of receiving an IABP preoperatively (203 with an IABP, 203 without an IABP). Clinical end points included total blood transfusion requirements, composite in-hospital morbidity and/or mortality, total hours in the intensive care unit, and length of hospital stay. Composite in-hospital morbidity consisted of stroke, deep sternal wound infection, respiratory failure, reoperation for bleeding, new postoperative renal failure, sepsis, vascular complications including dissection and limb ischemia, and postoperative myocardial infarction.

All statistical analyses were performed using SAS Version 9.3 (SAS Institute Inc., Cary, NC, USA). Data analysis was performed retrospectively. Propensity score matching was used to match preoperative IABP patients to controls (non-preoperative IABP patients) on several potentially confounding variables. Propensity score matching is an efficient alternative to matching on individual variables [2]. The probability of receiving a preoperative IABP (that is the propensity score) was calculated using a logistic regression model. Factors included in the model were: age, gender, body mass index, left ventricular ejection fraction (LVEF), preoperative creatinine, presence of comorbidities (cerebral vascular disease, diabetes mellitus, hypertension, smoking, hyperlipidemia, peripheral vascular disease, dialysis, congestive heart failure, chronic obstructive pulmonary disease), preoperative use of antiplatelet agents and/or anticoagulants including intravenous heparin, use of intravenous nitroglycerine, reoperation, time of between myocardial infarction and CABG, coronary artery disease burden (number of vessels with >70% stenosis, left main coronary artery disease >50%), concurrent valvular disease, and preoperative hemodynamic instability. Each patient was matched to a single control, based on the propensity score, using the SAS macro OneToManyMTCH [3]. Conditional logistic regression was then used to compare binary outcomes between the resulting matched pairs. The Wilcoxon signed-rank test was used to compare continuous outcomes between the matched pairs. The Mann-Whitney test was used to evaluate the comparability of continuous factors used to generate the propensity score between the two groups both prior to and after matching. Similarly, the chi-square test was used to evaluate the comparability of categorical factors.

## Results

Preoperative characteristics for the 877 patients undergoing CABG after an acute myocardial infarction are listed in Table 1. A total of 39.5% of patients received a preoperative IABP. Those receiving a preoperative IABP were younger with fewer comorbidities. In particular, patients with a preoperative IABP had less preoperative renal insufficiency, hypertension, cerebrovascular disease, and peripheral vascular disease. However, preoperative IABPs were placed more often in patients with hemodynamic instability, lower ejection fractions, left main coronary artery disease, and in patients on intravenous nitroglycerin. Patients with IABPs were more likely to undergo CABG on an emergent basis and within 24 hours of their myocardial infarction.

**Table 1:** **Pre-matched characteristics of patients**

	**No preoperative IABP (n = 531)**	**Preoperative IABP (n = 346)**	***P*** **value**
Age (years)	67.0 (60.0, 75.0)	64.5 (57.0, 74.0)	0.008
Female	145 (27.3%)	83 (24.0%)	0.274
Body mass index	27.6 (24.8, 31.2)	26.8 (24.4, 29.8)	0.076
Cerebrovascular disease	110 (20.7%)	54 (15.6%)	0.058
Peripheral vascular disease	73 (13.8%)	33 (9.5%)	0.062
Diabetes	273 (51.4%)	164 (47.4%)	0.245
Congestive heart failure	157 (29.6%)	109 (31.5%)	0.542
Dialysis	28 (5.3%)	9 (2.6%)	0.054
Preoperative creatinine	1.10 (0.90, 1.50)	1.05 (0.90, 1.30)	0.021
Chronic obstructive lung disease	163 (30.7%)	120 (34.7%)	0.217
Hypercholesterolemia	228 (42.9%)	155 (44.8%)	0.587
Hypertension	442 (83.2%)	241 (69.7%)	<0.001
Ejection fraction (%)	45 (35, 55)	40 (30, 48)	<0.001
Prior cardiac operation	9 (1.7%)	7 (2.0%)	0.723
Preoperative anticoagulation	357 (67.2%)	299 (86.4%)	<0.001
Preoperative antiplatelet	120 (22.6%)	77 (22.3%)	0.905
Preoperative intravenous nitroglycerin	38 (7.2%)	68 (19.7%)	<0.001
Operative status			
Elective/Urgent	525 (98.9%)	292 (84.4%)	<0.001
Emergent	6 (1.1%)	54 (15.6%)	
Preoperative myocardial infarction			
<6 hrs	1 (0.2%)	2 (0.6%)	
<24 hrs	10 (1.9%)	76 (22.0%)	<0.001
1-7 days	394 (74.2%)	250 (72.3%)	
8-21 days	127 (23.9%)	20 (5.8%)	
Preoperative hemodynamic instability	10 (1.9%)	41 (11.8%)	<0.001
Vessels diseased			
Left circumflex artery	411 (77.4%)	239 (69.1%)	0.006
Right coronary artery	407 (76.7%)	256 (74.0%)	0.370
Left anterior descending	469 (88.3%)	280 (80.9%)	0.002
Left main coronary artery	105 (19.8%)	137 (39.6%)	<0.001
Triple-vessel disease	308 (58%)	177 (51.2%)	0.046

Continuous factors are summarized by median (25^th^ percentile, 75^th^ percentile), categorical factors by frequency (percentage). IABP, intraaortic balloon pump.

No significant differences in demographics or preoperative risk factors were found between patients with and without an IABP after propensity score matching (Table 2). Patients with and without an IABP had similar rates of prior percutaneous coronary interventions (18.2% vs. 16.3%, *P* = 0.60) after propensity matching. Similarly, there was no statistically significant differences in the rate of percutaneous coronary interventions performed immediately before CABG (7.4% vs. 3.4%, *P* = 0.08). There was no difference in the use of antiplatelet and anticoagulation agents between the two cohorts after propensity matching (Table 2). In particular, there was no difference in the preoperative use of GPIIa/IIIb inhibitors (2.9% vs. 2.5%, *P* = 0.76). The modified STS predicted risks of mortality and combined morbidity and mortality were also comparable after propensity score matching (Table 2). Although the groups were matched only on preoperative characteristics, there were no major differences in the intraoperative characteristics between the two groups after matching (Table 3). Specifically, there were no statistically significant differences in percentage of off-pump cases, preoperative hematocrit, cross-clamp times, and cardiopulmonary bypass times. There was a statistically significant difference in the mean total number of distal anastomoses between the two cohorts (2.9 vs. 3.1) although the median number of anastomoses is 3.0 for both groups.

**Table 2:** **Post-matched characteristics of patients**

	**No preoperative IABP**	**Preoperative IABP**	***P*** **value**
	**(n = 203)**	**(n = 203)**	
Age (years)	66.0 (59.0, 74.0)	65.0 (57.0, 74.0)	0.514
Female	59 (29.1%)	47 (23.2%)	0.175
Body mass index	27.6 (24.2, 30.4)	26.9 (24.6, 30.5)	0.716
Cerebrovascular disease	32 (15.8%)	35 (17.2%)	0.688
Peripheral vascular disease	28 (13.8%)	24 (11.8%)	0.553
Diabetes	87 (42.9%)	103 (50.7%)	0.112
Congestive heart failure	55 (27.1%)	60 (29.6%)	0.582
Dialysis	8 (3.9%)	6 (3.0%)	0.587
Preoperative creatinine	1.00 (0.90, 1.30)	1.00 (0.90, 1.30)	0.953
Chronic obstructive pulmonary disease	69 (34.0%)	72 (35.5%)	0.755
Hypercholesterolemia	74 (36.5%)	75 (37.0%)	0.918
Hypertension	156 (76.9%)	158 (77.8%)	0.812
Ejection fraction (%)	40 (30, 50)	40 (30, 50)	0.914
Prior cardiac operation	5 (2.5%)	3 (1.5%)	0.475
Preoperative anticoagulation	167 (82.3%)	168 (82.8%)	0.896
Preoperative antiplatelet	54 (26.6%)	54 (26.6%)	1.000
Preoperative intravenous nitroglycerin	28 (13.8%)	29 (14.3%)	0.886
Operative status			
Elective/Urgent	197 (97.0%)	190 (93.6%)	0.100
Emergent	6 (3.0%)	13 (6.4%)	
Preoperative myocardial infarction			
<6 hrs	1 (0.5%)	0 (0.0%)	
<24 hrs	10 (4.9%)	11 (5.4%)	0.930
1-7 days	171 (84.2%)	172 (84.7%)	
8-21 days	22 (10.8%)	20 (9.9%)	
Preoperative hemodynamic instability	9 (4.4%)	8 (3.9%)	0.804
Vessels diseased			
Left circumflex artery	148 (72.9%)	148 (72.9%)	1.000
Right coronary artery	144 (70.9%)	157 (77.3%)	0.141
Left anterior descending	171 (84.2%)	170 (83.7%)	0.892
Left main coronary artery	65 (32.0%)	59 (29.1%)	0.518
Triple-vessel disease	104 (51.2%)	113 (55.7%)	0.371
Modified STS mortality risk	2.1% (1.1, 5.2)	1.9 (1.0, 4.1)	0.354
Modified STS morbidity/mortality risk	18.8% (10.7, 31.4)	18.1% (11.1, 27.2)	0.350

Continuous factors are summarized by median (25^th^ percentile, 75^th^ percentile), categorical factors by frequency (percentage). IABP, intraaortic balloon pump; STS, Society of Thoracic Surgeons.

**Table 3:** **Intraoperative characteristics of matched patients**

	**No preoperative IABP**	**Preoperative IABP**	***P*** **value**
Preoperative hematocrit	38.0 (34.0, 42.0)	36.5 (33.0, 42.0)	0.532
Off pump (%)	24 (11.8%)	20 (9.8%)	0.528
Cardiopulmonary bypass time (min)	97.0 (75.0, 134.0)	108.0 (80.0, 130.5)	0.580
Aortic cross-clamp time (min)	56.0 (43.0, 73.0)	58.0 (46.0, 71.0)	0.224
Lowest temperature on bypass (°C)	32.0 (28.0, 34.0)	32.0 (28.0, 34.0)	0.444
Number of arterial grafts	1.0 (1.0, 1.0)	1.0 (1.0, 1.0)	0.611
Number of distal anastomoses	3.0 (2.0, 4.0)	3.0 (3.0, 4.0)	0.045

Continuous factors are summarized by median (25^th^ percentile, 75^th^ percentile), categorical factors by frequency (percentage). IABP, intraaortic balloon pump.

Differences in postoperative morbidity and intensive care unit length of stay were observed between the two propensity-matched cohorts (Table 4). Patients with an IABP were more likely to have the composite end point of in-hospital morbidity (24.1% vs 12.8%, odds ratio: 2.2, 95% confidence interval: 1.3 to 3.8, *P* <0.004), and increased hours in the intensive care unit (median hours: 69.0 vs. 46.0, *P* <0.013) as compared to a propensity-matched cohort without an IABP. There was no difference in the overall length of hospital stay. Postoperative complications in the two matched cohorts are summarized in Table 5. Out of the propensity-matched patients, patients with a preoperative IABP had higher rates of prolonged ventilation (22.2% vs. 9.4%), renal failure requiring dialysis (2.0% vs. 0.5%), and gastrointestinal complications (2.5% vs. 1.0%) as compared to patients without an IABP. Similarly, there was increased deep sternal wound infection (2.0% vs. 1.0%) and septicemia (1.5% vs. 0.5%) in patients with a preoperative IABP as compared to those without. In-hospital mortality was 2.5% with a preoperative IABP and 1.0% without a preoperative IABP. Placement of an IABP was not associated with increased vascular complications.

**Table 4:** **Differences in outcome of matched patients**

	**No IABP**	**IABP**	***P*** **value**
Complications n (%)	26 (12.8%)	49 (24.1%)	<0.004
ICU stay (hours)^*^	46.0 (26.0, 79.0)	69.0 (44.8, 103.0)	<0.013
Hospital LOS (days)^*^	6.0 (5.0, 9.0)	7.0 (5.0, 9.0)	0.245

^*^Median (25^th^ percentile, 75^th^ percentile). ICU, intensive care unit; LOS, length of stay.

**Table 5:** **Description of complications of matched patients**

**Complication**	**No preoperative**	**Preoperative**
	**IABP (n = 203)**	**IABP (n = 203)**
Stroke	2 (1.0%)	2 (1.0%)
Deep sternal wound infection	2 (1.0%)	4 (2.0%)
Respiratory insufficiency	19 (9.4%)	45 (22.2%)
Reoperation for bleeding	3 (1.5%)	2 (1.0%)
Renal failure requiring dialysis	1 (0.5%)	4 (2.0%)
Gastrointestinal complications	2 (1%)	5 (2.5%)
Septicemia	1 (0.5%)	3 (1.5%)
Lower extremity vascular complications	2 (1.0%)	1 (0.5%)
Mortality	2 (1.0%)	5 (2.5%)

IABP, intraaortic balloon pump.

Patients with a preoperative IABP also had higher perioperative transfusion requirements as compared to non-IABP patients (Table 6). The two groups were comparable in their preoperative antiplatelet and anticoagulation therapy. Preoperative clopidogrel was administered in 52.2% and 54.2% of patients with and without a preoperative IABP, respectively (*P* = 0.69). Similarly, 86.2% of patients with and 86.7% of patients without a preoperative IABP were on preoperative aspirin (*P* = 1.00). Compared to patients without an IABP, a higher percentage of patients with a preoperative IABP required perioperative transfusions of packed red blood cells (82.3% vs. 67.5%), platelets (48.8% vs. 35.5%), and fresh frozen plasma (25.6% vs. 14.8%) (Table 6). Cryoprecipitate transfusion requirements were not statistically different between the two groups. Not only were patients with a preoperative IABP more likely to receive packed red blood cell transfusions, they also required more units of red blood cells (3.6 units vs. 2.7 units, *P* <0.001).

**Table 6:** **Transfusion requirements of matched patients**

	**No IABP (n = 203)**	**IABP (n = 203)**	***P*** **value**
Packed red blood cells	137 (67.5%)	167 (82.3%)	<0.001
Platelets	72 (35.5%)	99 (48.8%)	0.009
Fresh frozen plasma	30 (14.8%)	52 (25.6%)	0.001
Cryoprecipitate	12 (5.9%)	21 (10.3%)	0.145

IABP, intraaortic balloon pump.

Although patients with a preoperative IABP were more likely to present with ST-elevation myocardial infarctions (32.5% vs. 12.8%, *P* <0.01), there was no association between the presence of ST-elevation myocardial infarction and postoperative outcomes. Inclusion of the presence of ST-elevation myocardial infarction into the propensity matching model reduced the number of matched pairs from 203 to 169 but did not change the overall results. As compared to patients without a preoperative IABP, patients with a preoperative IABP still had increased composite outcome of morbidity and mortality (24.2% vs. 14.2%, *P* = 0.02) and increased length of intensive care unit stay (median: 67 hours vs. 47 hours, *P* <0.01). Similarly, patients with a preoperative IABP had increased packed red blood cell (*P* <0.01), fresh frozen plasma (*P* = 0.03), and platelet (*P* <0.01) transfusions as compared to patients without an IABP even after adjusting for the presence of ST-elevation myocardial infarction in the propensity matching model.

## Discussion

Current recommendations for the use of IABP in patients undergoing CABG are predominantly based on early small randomized studies by Christenson *et al.* [4–8]. These studies collectively found benefits to the preoperative placement of an IABP in high-risk patients undergoing CABG. Patients included in the high-risk category included those with a triad of triple-vessel disease, arterial hypertension and LVEF <40% and/or patients undergoing CABG with at least two of the following: LVEF <40%, unstable angina, left main stenosis >70%, or reoperation. Contemporary studies have challenged the effectiveness of a preoperative IABP in patients undergoing CABG, even in high-risk cohorts [9–12]. Concurrent with this, there is increasing evidence demonstrating the lack of mortality benefit for the insertion of an IABP in patients with acute myocardial infarction and/or cardiogenic shock [13,14].

Despite its wide acceptance and use, recommendation for use of IABPs in the treatment of cardiogenic shock was currently downgraded from a class I recommendation to a class IIa and IIb recommendation in the American and European guidelines, respectively [15–17]. The IABP-SHOCK II trial was designed to validate such recommendations. It randomized 600 patients with acute myocardial infarction complicated by cardiogenic shock to receive an IABP or not. All patients were expected to undergo early revascularization via either percutaneous coronary intervention or CABG. The trial found that the use of an IABP did not significantly reduce 30-day morbidity or mortality in this patient population [13]. Long-term follow up at six months and one year after infarct continued to show no significant differences in mortality, re-infarction, recurrent revascularization, and quality-of-life measures between the two groups, thus questioning the role of IABPs in patients with acute myocardial infarction and cardiogenic shock [14]. The observed trend toward a higher rate of implantation of ventricular assist devices in the control group, which provide greater hemodynamic and cardiac support than IABPs, may have attenuated differences in outcomes between controls and patients with an IABP.

The role of IABPs in patients with acute myocardial infarction without cardiogenic shock has also been challenged. Propensity-matched analysis comparing patients undergoing primary percutaneous coronary intervention for myocardial infarction without cardiogenic shock showed similar in-hospital mortality rates between those that received an IABP at the time of revascularization as those that did not. The study also found that the IABP group had significantly more bleeding complications resulting in greater transfusion requirements [18]. The Counterpulsation to Reduce Infarct Size Pre-PCI Acute Myocardial Infarction (CRISP AMI) trial randomized patients with acute anterior ST-segment elevation myocardial infarction without cardiogenic shock to placement of an IABP prior to percutaneous coronary intervention (PCI) or primary PCI alone. The study found that placement of an IABP prior to PCI did not result in reduced infarct size or differences in clinical outcomes at six months as compared to PCI alone in this patient population [19].

There is currently no consensus on the use of preoperative IABPs in patients undergoing CABG. Christenson *et al*. reported in a single-center randomized controlled trial that the use of preoperative IABPs in patients undergoing CABG surgery with any two of the following: LVEF <30%, left main stenosis >70%, reoperation, or unstable angina decreased morbidity and mortality when compared to not using a preoperative IABP [8]. Subsequent studies have demonstrated the efficacy of a preoperative IABP in hemodynamically stable, high-risk patients undergoing CABG and in patients undergoing reoperative CABG [4,5,7,20]. In contrast, a large multi-center study utilizing propensity-matching analysis by Baskett *et al*. failed to show clinical benefit in the preoperative use of IABPs in patients undergoing CABG [10]. Rather, the study showed that the use of preoperative IABPs was associated with higher mortality. Surprisingly, the 1.5- to 3-fold increase in mortality rates in patients with a preoperative IABP persisted in subgroup analysis of high-risk patients including those with left main coronary artery disease, reoperations, LVEF <40%, unstable angina, and prior myocardial infarctions.

IABPs are widely used to temporize patients waiting for CABG surgery after an acute myocardial infarction, yet the impact of IABPs on clinical outcomes in this patient population is uncertain. This study found that the use of a preoperative IABP in patients undergoing isolated CABG after acute myocardial infarction is associated with increased in-hospital morbidity and longer postoperative intensive care unit stay. Interestingly, although the most reported major complication from IABPs is limb ischemia and vascular injury, the use of preoperative IABPs in our study was not associated with increased rates of such complications. This may be secondary to the use of smaller sheath sizes and the increasing use of sheathless insertion techniques. Rather, the use of preoperative IABPs in our study is associated with increased infectious complications such as deep sternal wound infection and septicemia and end organ dysfunction such as respiratory, gastrointestinal, and renal insufficiency. Infection and embolic events are known complications of IABPs [9,21]. However, increased end organ dysfunction has not been directly attributable to the correct use and positioning of IABPs.

Our study further showed that patients with an IABP had increased intraoperative and postoperative transfusion requirements. Aside from the presence of an IABP, the matched groups had similar risk factors for transfusion requirements. Specifically, the two groups were comparable in age, body mass index, preoperative hematocrit, use of preoperative antiplatelet agents, cross-clamp and bypass times, and degree of hypothermia on bypass. The results of this study are congruent with other studies that demonstrate the presence of an IABP as an independent risk factor for bleeding and increased transfusions [22]. Patients with an IABP may have increased risk for blood transfusions as they are not only at risk for vascular injury and/or bleeding at the access site, but they are also at increased hemorrhagic risk secondary to platelet consumption and dysfunction and increased fibrinolysis secondary to prolonged exposure of blood components to the synthetic surface of the balloon. The association between perioperative transfusions and increased in-hospital morbidity and mortality in cardiac surgery patients has been well described [23,24]. Transfusions have been associated with increased infectious, renal, respiratory, cardiac, and neurologic complications and decreased long-term survival after cardiac surgery [23–26]. In particular, perioperative blood transfusions have been associated with acute lung injury, pulmonary dysfunction, and increased time to extubation in postoperative cardiac surgery patients [23,27–29]. The extent to which the increased morbidity and mortality that is seen in the IABP cohort, including increased end organ dysfunction and pulmonary insufficiency, can be attributed to increased transfusion requirements cannot be elucidated from this study and requires further investigation. It may be possible that the hemodynamic and myocardial protective benefits of IABPs are attenuated by the well-described negative effects of increased blood transfusions on clinical outcomes.

As with all studies utilizing propensity score matching, this study is limited by the assumption that all covariates are accounted for in the matching. This study incorporates the majority of known preoperative variables that may influence the decision for insertion of preoperative IABPs in the propensity score matching in order to reduce the effect of such confounders. Additionally, as with all observational studies, this study is subject to misclassification of comorbid status. This may affect the results if there are significant differences in the prevalence of comorbidities between patients with a preoperative IABP and those without. The use of validated state audited data obtained for the New York State Cardiac Surgery Reporting System in this study rather than reliance on administrative codes reduces the risk of misclassification. Furthermore, the use of marker-based variables that are not as vulnerable to misclassification such as age, creatinine, ejection fraction, time from myocardial infarction, and number of vessels diseased in the propensity model decreases the inaccuracy of comparing different populations using imperfectly measured confounders. Despite propensity matching, it is possible that the IABP cohort remains a higher risk cohort as evidenced by the higher rate of emergent surgery and longer operative times. Such differences, although statistically insignificant, may have an impact on clinical outcomes. The similarities in the modified calculated STS scores between the two cohorts would, however, suggest that the two groups were adequately matched with respect to accepted risk stratification models. Another limitation of this study is that patients at extremes of risk were eliminated after propensity matching. The matched cohort had less use of preoperative intravenous nitroglycerin, less emergent operations and less preoperative hemodynamic instability as compared to the unmatched group with preoperative IABP. Therefore, the results of this study may not be applicable to extremely high-risk patients. Another limitation of the study is that the two groups were not matched on the incidence of ST-segment elevation myocardial infarction and non-ST-segment myocardial infarction. Although a greater percentage of patients with a preoperative IABP sustained ST-segment elevation myocardial infarctions, the differences in outcomes between patents with and without a preoperative IABP persisted even after subgroup propensity matching analysis matching patients on the presence of ST-segment elevation versus non-ST-segment myocardial infarction. This study was also limited to patients undergoing isolated CABG. Patients requiring concomitant cardiac surgical procedures and/or those with mechanical complications of acute myocardial infarction such as acute mitral regurgitation or myocardial rupture were excluded from this study as the pathophysiology of their cardiac disease is different from patients requiring isolated CABG. The results of this study cannot, therefore, be extended to those patient populations. Lastly, the decision to insert a preoperative IABP was at the discretion of the surgeon and/or cardiologist, and the indications for insertion were not captured. This study is therefore unable to identify differences in clinical outcomes based on specific indications for IABP placement.

## Conclusions

The benefits of a preoperative IABP in patients undergoing isolated CABG surgery after an acute myocardial infarction remain uncertain. Further studies to identify specific subpopulations that will benefit from a preoperative IABP and stricter guidelines for the indications for placement are warranted. In addition, focused efforts to limit blood transfusions after placement of an IABP may impact the effect of the IABP on clinical outcomes.

## Key messages

- Placement of a preoperative IABP in patients undergoing CABG after sustaining a myocardial infarction may increase the patient’s risk of perioperative transfusions and postoperative morbidity
- Studies to define the specific subpopulation of patients that would benefit from a preoperative IABP prior to CABG are warranted

## Abbreviations

CABG: coronary artery bypass grafting
IABP: intraaortic balloon pump
LVEF: left ventricular ejection fraction
PCI: percutaneous coronary intervention
STS: Society of Thoracic Surgeons
